# Patient’s and the Therapist’s Attachment Representations, Attachment to Therapists, and Self-Esteem-Change Through Psychotherapy

**DOI:** 10.3389/fpsyg.2021.711296

**Published:** 2021-11-02

**Authors:** Katja Petrowski, Hendrik Berth, Peter Beiling, Vanessa Renner, Thomas Probst

**Affiliations:** ^1^Department of Psychosomatic Medicine and Psychotherapy, University Medical Center of the Johannes Gutenberg-University, Mainz, Germany; ^2^Department of Psychotherapy and Psychosomatic Medicine, University Hospital Carl Gustav Carus Dresden, Technical University Dresden, Dresden, Germany; ^3^Medical Psychology and Medical Sociology, University Hospital Carl Gustav Carus Dresden, Technical University Dresden, Dresden, Germany; ^4^Department for Psychotherapy and Biopsychosocial Health, Danube University Krems, Krems, Austria

**Keywords:** attachment, self-esteem, psychotherapy, mental health, adult attachment

## Abstract

**Objectives:** The present naturalistic study aims to investigate the differential effects of the patient’s and the therapist’s attachment representations on the attachment to the therapist as perceived by the patient, and their impact on self-esteem-change through psychotherapy.

**Methods:** Attachment variables of *N* = 573 patients as well as *N* = 16 therapists were assessed. Attachment representations were measured for therapists and patients via the Bielefelder Questionnaire for Client Attachment Exploration, the Relationship Specific Attachment to Therapist Scales and the Adult Attachment Interview. The patient’s attachment to therapists was evaluated and patients’ self-esteem was measured via the Frankfurter Selbstkonzeptskalen at the beginning and end of psychotherapy.

**Results:** Although there were significant effects of the patient’s attachment representations on the perceived attachment to the therapist as well as between the perceived attachment to the therapist and the amount of self-esteem-change, the therapist’s attachment style had no significant influence on the perceived attachment to the therapist.

**Conclusion:** Self-esteem-change through psychotherapy is influenced by the actually formed attachment relationship as perceived by the patient. The patient’s attachment representations but not the therapist’s attachment style contributes to the actual patient’s attachment to the therapist.

## Introduction

A growing body of research suggests that the attachment theory provides an important framework for understanding the etiology of mental disorders, the patients’ attachment to the therapist as well as patient’s changes due to psychotherapy (Bowlby, 1973, 1982; Belsky and Cassidy, 1994; Mallinckrodt et al., 1995; Eames and Roth, 2000; Parish and Eagle, 2003; Sauer et al., 2003). The psychological disorders differ in their attachment specific etiology as well as in their specific psychological symptoms (Buchheim et al., 2002). In depression, social anxiety disorder and several other disorders a low self-esteem is a general symptom (Borras et al., 2009; Vater et al., 2010; Sowislo and Orth, 2013) which can be explained by the attachment representation (Brennan and Morris, 1997). Therefore, attachment styles play an important role in different disorders and might even influence therapy outcome. However, it is still unknown whether the attachment theory can also explain the patient-therapist alliance during psychotherapy and the changes in self-esteem over time.

Attachment research has focused extensively on how individual differences in attachment relate to functioning in romantic relationship and therapeutic alliance (Collins et al., 2006). Hazan and Shaver (1994) translated the attachment theory of early childhood (Bowlby, 1973; Ainsworth, 1985) into an attachment theory for adults, romantic love. Based on this concept, Collins and Read (1990) attempted to measure attachment behavior in adults and to analyze this factor analytically. Three underlying dimensions crystallized the degree of closeness with which an individual feels comfortable, the degree of trust that the individual can bring to others, and the degree of anxiety the person feels about being left or being abandoned unloved (Collins and Read, 1990). Later on, Bartholomew and Horowitz (1991) proposed a two-dimensional model which bases on the positive-negative self-perception and perception of others. These dimensions reflect differences in coping strategies with unsatisfied needs of closeness in the early childhood (Bartholomew, 1990) and were used in other models as well with the dimensions secure-fearful and independent-dependent (Asendorpf et al., 1997).

As the attachment system in adulthood is believed to be activated at times of threat or distress, this may be the time many people seek therapeutic help, which led researchers to conceptualize the therapeutic relationship in terms of attachment (Slade, 2008). Therefore, the attachment model was transferred and used as bases for the expectations of the patient toward the therapist concerning the psychotherapeutic relationship (Höger, 1999). Hereby the attachment styles were described on the three scales “readiness to self-disclosure,” “problems with feeling accepted” and “need for care” (Höger, 1999). The ambivalent-clinging patients are characterized by strong problems with feeling accepted, high need for care, and mediocre readiness to self-disclosure. The ambivalent-withdrawn patients show strong problems with feeling accepted, a low need for care, and high readiness to self-disclosure. The avoidant-withdrawn patients display fewer problems with feeling accepted, low need for care, and low readiness to self-disclosure. Secure attached patients demonstrate low problems with feeling accepted, a low need for care, and high readiness to self-disclosure. In contrast, the conditioned secure attached patients are characterized by a lower need for care.

Several studies have shown that attachment styles of patients as well as attachment styles of therapists can influence therapeutic alliance and psychotherapy outcome (Strauss et al., 2006; Smith et al., 2010; Degnan et al., 2016; Reiner et al., 2016; Levy et al., 2018; Levy and Johnson, 2019). Numerous studies with different patient populations, treatment modalities as well as therapeutic approaches have shown that patients’ attachment characteristics and attachment related interpersonal expectations predict treatment outcome in psychotherapy (Levy et al., 2011). Since attachment states of mind influence emotion regulation processes, the therapists’ attachment states of mind also have an effect on the psychotherapeutic process and outcome (Levy and Kelly, 2010; Slade and Holmes, 2019), the ability to develop an intimate therapeutic relationship (Mallinckrodt, 2000), to handle alliance ruptures and to manage counter-transference in the therapeutic relationship (Ligiéro and Gelso, 2002; Mohr et al., 2005; Slade and Holmes, 2019). Romano et al. (2009) have even shown that the therapist’s attachment representation influences the patient’s experienced attachment to the therapist. Interestingly, a dissimilarity in attachment representations of patient and therapist led to better alliances and a higher functioning of patients (Tyrrell et al., 1999). Also, therapist’s personal attributes (e.g., openness, honesty, and respectfulness) and techniques (e.g., reflection and noting past therapy success) were found to have a positive impact on the alliance between therapist and patient as well as on therapy outcome (Ackerman and Hilsenroth, 2003). As Degnan et al. (2016) showed, some studies found therapist attachment styles and interactions between therapists’ and patients’ attachment styles contribute to alliance and therapy outcome. Whereas anxiously attached therapists established poorer working alliances with their patients, therapist attachment security was related to improved therapy outcomes (Degnan et al., 2016). However, findings suggest that these relationships may not be straightforward and that attachment styles of therapists and patients interact in influencing the alliance and therapy outcomes (Degnan et al., 2016). The described findings were not consistent across studies, but mixed results were found in studies on therapist attachment and its influence on therapy process and outcome. Also, other reviews emphasized methodological weaknesses of studies reviewed, such as patients’ selection criteria and high variabilities between studies, for example because of different treatment approaches or implemented instruments (Ackerman and Hilsenroth, 2003; Lingiardi et al., 2018).

One mechanism by which attachment might develop and change involves the formation of self-concept and self-esteem (Cassidy, 1990). The self-concept underlying attachment reflects the sense of lovability or being worthy of love (Bowlby, 1973; Bartholomew and Horowitz, 1991). Based on Bowlby’ s mental model, Hazan and Shaver (1994) stated that “attachment theory thus implies that beliefs and feelings about the self, especially social and global self-esteem, are determined in part by the responsiveness of the caregiving environment (p. 5).” Consistent with their conclusion, several studies have found that higher levels of global self-esteem are reported by respondents who described themselves as secure or dismissing (positive self-models) than by those who described themselves as preoccupied or fearful (negative self-models; Collins and Read, 1990; Bylsma et al., 1997; Mickelson et al., 1997). Depressive patients with more insecure attachment style showed a low global self-esteem (Fuhr et al., 2017) and participants with secure attachment style reported higher global self-esteem than participants with a preoccupied or fearful attachment style (Bylsma et al., 2010). The positivity of an individual’s attachment self-model was highly related to the positivity of their self-concept (Griffin and Bartholomew, 1994).

Concerning the stability of self-esteem, higher attachment anxiety relates to more unstable self-esteem (Foster et al., 2007). In specific, defensiveness and self-aggrandizing tendencies are associated with unstable (in particular high) self-esteem (Kernis, 2003) and absent with secure attachment (Mikulincer et al., 2005). Attachment security is the most important determinant of authentic, stable self-worth (Foster et al., 2007). A relationship between the stability of self-esteem and attachment avoidance could not be found (Srivastava and Beer, 2005; Foster et al., 2007).

Based on these findings it seems possible that also in the relationship between therapist and patient, self-esteem could be influenced by attachment styles of therapists and patients. Past studies showed a mediating role of low self-esteem in the relation between attachment style and depressive symptoms (Fuhr et al., 2017). Meta-analyzes showed a positive effect of psychotherapy on self-esteem as an outcome, whereas psychotherapeutic interventions did lead to an increase in self-esteem compared to an untreated control group (Smith and Glass, 1977; Linardon et al., 2019). The improvement in self-esteem is even higher than the improvement in general symptom reduction and general capability (Smith and Glass, 1977). Even though these meta-analyzes showed changes in self-esteem through psychotherapy, the possible moderating role of the attachment of patient and therapist has not yet been taken into account. The present study sought to fill this gap and addressed the following research questions:

Regarding attachment representations of patients and therapists, based on the attachment theory of Bowlby (1982) and findings by Höger (1999) and Tyrrell et al. (1999), we hypothesize that:

(1) Patients’ anticipated readiness for self-disclosure positively and patients’ anticipated problems in feeling accepted negatively predict the amount of the patients’ perceived attachment to the therapist as secure (H1).
(2) Anticipated need for care of the patient positively predicts the amount of the patients’ perceived attachment to the therapist as dependent (H2).
(3) Highly preoccupied therapists form attachment perceived as less dependent by the patient as well as highly insecure therapists form attachment perceived as more dependent by the patient (H3).

Regarding self-esteem as an important outcome of psychotherapy (Smith and Glass, 1977) and its relation to attachment characteristics (Feeney and Noller, 1990; Bylsma et al., 1997; Foster et al., 2007), we assume that:

(4) High levels of security in the patients’ attachment to the therapist lead to greater changes in self-esteem (H4).

## Materials and Methods

### Participants

The present study analyzed data of *N* = 573 patients, who were treated by *N* = 16 psychotherapists at the Carl Gustav Carus University Hospital Dresden between 2005 and 2013, including inpatient as well as day patient care. The original sample consisted of 1.198 patients who were treated in the clinic within 2 years. As data was only collected for 1 year, the attachment style of the therapists and patients as well as attachment of patients to therapists were assessed only for a subsample of *N* = 573 patients.

The patients’ ages varied between 18 and 77 (*M* = 37.69; SD = 13.11), 66.3% were female. 29.7% of the participants were working full-time, 9.6% received disability pension. 49.7% of the participants were single, 27.7% were married, and 13.3% were divorced. The average duration of treatment was 56 calendar days (SD = 31.30). Patients saw their primary therapist for individual focal therapy at least once a week for 50 mins. Regarding the primary ICD-10 diagnoses confirmed by SCID [Spitzer et al. (1992); German version by Wittchen et al. (1997)], most patients were diagnosed with affective disorders (F30–F39; 56%), followed by anxiety disorders (F40–F49; 11%). 128 participants had a total of four diagnosis, four participants met a maximum of eight diagnosis. The general symptom severity at admission was quite high (SCL-90-R-GSI T value at admission was *M* = 70.04; SD = 10.75) and had decreased significantly at discharge even though the level was still high [*M* = 60.85, SD = 13.88; *t*(557) = 19.48, *p* < 0.001]. Depressive symptoms of the participants reflected a moderate depressive episode (*M* = 21.67, SD = 11.18) at admission and a marginal depressive episode at discharge (*M* = 13.94, SD = 12.05).

The *N* = 16 psychotherapists were members of the staff of the Carl Gustav Carus University Hospital in Dresden, Germany from 2005 to 2013. Their ages varied between 26 and 60 (*M* = 43.94, SD = 9.68), 10 of them were female. They were psychologists or physicians with a psychotherapeutic specialization (*n* = 9 clinical psychologists and *n* = 7 physicians). Their clinical experience ranged from 2 to 33 years (*M* = 9.94, SD = 9.68) with three different therapeutic orientations (44% psychodynamic, 37% cognitive-behavioral, and 19% systemic/family therapy). Every one of the therapists treated from 2 to 121 patients (*M* = 35.81; SD = 38.82) who were assigned to them at random.

### Instruments

The Bielefelder Questionnaire for Client Attachment Exploration (BFKE; Höger, 1999; Pollak et al., 2008) and the Relationship specific Attachment to Therapist (BBE; Asendorpf et al., 1997) were used to assess attachment style. The BFKE assesses self-reported attachment or rather relationship expectations toward the therapist with 33 items on three scales: “readiness for self-disclosure,” “problems with feeling accepted,” and “need for care.” The BFKE was constructed based on attachment theories by Bowlby (1975), Ainsworth (1985), and Main (1990). The three scales can be used for classification based on one of five factors, which can be interpreted as attachment. The stability of the subscales could be confirmed in several studies. The three scales show internal consistencies (Cronbachs Alpha) of α = 0.83–0.84 (Höger, 1999; Pollak et al., 2008). The BBE comprises a total of 36 items and is based on Bartholomew’s model of attachment styles in adulthood (Bartholomew and Horowitz, 1991). It is intended to measure the quality of attachment to important caregivers in adulthood using the axes “secure-fearful” and “dependent-independent.” High values reflect high security and high dependence. The BBE show consistencies (Cronbach’s alpha) of α = 0.71–0.87 (Asendorpf et al., 1997).

The Frankfurter Selbstkonzeptskalen (FSKN; Deusinger, 1986) is a self-report questionnaire consisting of 78 items. The FSKN is used for determining the respective image or self-concept that the individual has developed in important areas of the self. The scales are intended to capture a system of attitudes (in the sense of attitudes) to one’s own person, which are interpreted as aspects of the “identity” of the person. It is used to measure the self-esteem change from admission to discharge. The items load on 10 subscales: general performance (FSAL), general problem solving (FSAP), behavioral and decision-making security (FSVE), self-esteem (FSSW), sensitivity and mood (FSEG), resistance to groups with significant other people (FSST), social contact and sociability (FSKU), appreciation from others (FSWA), irritability from others (FSIA) and feelings and relationships with others (FSGA). Internal consistencies (Cronbachs alpha) of the 10 subscales varied between α = 0.93 and 0.97 (Deusinger, 1986).

The Adult Attachment Interview (AAI; Main and Goldwyn, 1985, 1996; George et al., 2001) is a semi-structured interview with 18 questions. The individual is encouraged to present own thoughts, feelings, and memories regarding early attachment experiences. The AAI assesses the individual’s current attitude toward attachment in terms of the past and the present and the extent to which an individual is capable of spontaneously telling childhood history in a cooperative, coherent and plausible way. The interviews are transcribed to analyze the individual’s organization of speech employing an analytical discourse technique regarding attachment (Grice, 1993). The result of the content analysis can then be classified into one of the four following categories (Main and Goldwyn, 1996): secure/autonomous (F), insecure/dismissing (Ds), insecure/preoccupied (E), and disorganized/unresolved trauma (U). The AAI has excellent psychometric properties (Hesse, 1999, 2008) showing high test-retest stability after 3 months (kappa = 0.79; van Ijzendoorn, 1995) and after 18 months (kappa = 0.73; Crowell et al., 1996).

In their overview of the first 10.000 AAIs in clinical and non-clinical groups Bakermans-Kranenburg and van Ijzendoorn (2009) suggested that dimensional approaches might be a fruitful perspective in attachment research (see also Bakermans-Kranenburg and van Ijzendoorn, 1993; Coppola et al., 2006; Schauenburg et al., 2010). A well-established dimensional approach for describing attachment representation was developed by Treboux et al. (2004). Hereby the “secure vs. insecure” and the “dismissing vs. preoccupied” were discriminant analytically distinguished (Treboux et al., 2004). In reference to the standardized values of a large sample a score higher than 0.00 on the secure vs. insecure scale represents an insecure attachment orientation. A score higher than 0.01 on the dismissing vs. preoccupied represents a preoccupied attachment orientation.

### Procedure

Patients completed the BFKE and the FSKN at admission and the BBE and the FSKN at discharge. To ensure a good database, only those patients with complete data sets available (regarding the questionnaires used) were included in the analysis resulting in the final sample of *N* = 573.

After the therapists were informed about the purpose of the study and consented to participate, the interviewers, who were completely unfamiliar with the patients and the therapists, administered the Adult Attachment Interview (AAI). The audio-taped interviews were assigned a study code and transcribed based on the audio-tapes. The AAI transcripts were coded by two independent reliable certified judges (who had been certified by Professor Mary Main and Professor Eric Hesse from the University of California, Berkeley, CA, United States at the AAI Institute in 1998 and for renewed reliability in 2007 by Professor Carol George from Mills College, Oakland, CA, United States). The inter-reliability between the two coders achieved a kappa of 0.82 in 20% of the AAIs. In case of a discrepancy between the two AAI raters, a consensus was found between them. The final dataset included the clinical data of the patients treated by the therapists, the therapists’ attachment scales (AAI Waters scale; Waters et al., 2002) as well as the personal characteristics of the therapists.

### Statistical Procedure

In order to investigate the hypotheses of the present study, the residualized change score of the self-esteem subscale of the FSKN was calculated via a linear regression in which self-esteem scores in the beginning predicted self-esteem scores in the end. In the first model, the three subscales of the BFKE (“readiness for self-disclosure,” “problems in feeling accepted,” and “need for care”) were used as predictors of the BBE dimensions (“secure-fearful” and “dependent-independent”; H1 and H2). Moreover, the secure-fearful dimension of the BBE was set to predict the amount of self-esteem change (self-esteem pre-post difference; H4). Additionally, the standard scores for the AAI Waters scales (“security-insecurity” and “dismissing-preoccupied”; Waters et al., 2002) were calculated and set to predict the BBE dimensions (H3).

The statistical analyses were carried out with Amos 20 using the following model fit indices: the minimum discrepancy divided by its degrees of freedom (CMIN/DF), the root mean square error of approximation (RMSEA), the comparative fit index (CFI), the normed fit index (NFI), the standardized root mean square residual (SRMR), and the Tucker Lewis Index (TLI). To compare the models, the Akaike information criterion (AIC) and the expected cross validation index (ECVI) were estimated. For a good model fit, the ratio CMIN/DF should be as small as possible (Arbuckle, 2009), and the CFI, NFI, and TLI should be higher than 0.95 (Schermelleh-Engel et al., 2003) whereby values greater than 0.90 are usually interpreted as indicators for an acceptable fit (Arbuckle, 2009). Furthermore, SRMR values smaller than 0.05 as well as RMSEA values smaller than 0.06 indicate a good model fit, and values smaller than 0.08 still reflect an adequate fit (Arbuckle, 2009). When comparing models, the model with the lower AIC and ECVI should be preferred. The models were estimated with the full maximum likelihood method.

## Results

The socio-demographic characteristics of the patients’ sample are given in Table 1.

**TABLE 1:** Socio demographic variables of the sample.

	*N* = 573
Age, years M (SD)	37.69 (13.11)
Age range (min–max)	18–77
**Sex**	**66.3% Female**
**Relationship status, *n* (%)**	
Married	168 (29.7)
Living separated from partner	27 (4.8)
Unmarried	285 (50.4)
Divorced	76 (13.5)
Widowed	9 (1.6)
**Education, *n* (%)**	
No school diploma	2 (0.4)
Still in school	4 (0.7)
Special needs school	6 (1.1)
Elementary school diploma	42 (7.5)
Middle school diploma	281 (49.9)
High school diploma	215 (38.2)

For the AAI-classification, 63% of the therapists showed an insecure and 37% a secure attachment representation. 56% of them showed a preoccupied and 44% a dismissive attachment representation. For the details about the distribution of the therapists’ attachment on the Waters AAI-scales, see Table 2. On average, the patients rated the attachment to the therapists as moderately secure and independent as shown in Table 2.

**TABLE 2:** Distribution of Adult Attachment Interview scales of therapists (*N* = 16) and distribution of the Relationship-specific attachment scales (BBE) of the patients (*N* = 573).

AAI-scale	Raw			Standardized		
	M	SD	Range	M	SD	Range
**Therapists**						
Secure vs. insecure	0.03	1.11	−2.36 to 1.51	0.02	1.19	−2.36 to 1.59
Dismissing vs. preoccupied	−0.13	1.01	−2.06 to 1.40	0.06	1.15	−2.02 to 1.76

	**M**			**SD**		**Range**

**BBE-scales: Patient attachment to the Therapist**						
BBE insecure-secure	3.80			0.75		1.5–5
BBE independent-dependent	2.30			0.63		1–4.5

The overall mean increase in self-esteem from pre- to post-treatment was *M* = 4.42 (SD = 8.85) and this change was statistically significant [*t*(572) = −11.95; *p* < 0.001]. Calculating a linear regression in which baseline self-esteem scores predicted outcome self-esteem scores, the residualized change score resulted in β = 0.595, *p* < 0.001. Regarding the self-esteem-change, there were neither significant differences between the therapists [*F*(15;557) = 1.01, *p* = 0.45] nor due to the experience of the therapists [*F*(1;571) = 2.08, *p* = 0.15] nor due to the theoretical orientation [*F*(5;567) = 0.468, *p* = 0.18].

The patients’ symptom severity at time of intake did not correlate significantly with the therapists’ attachment scales (Pearson Product Correlation Coefficient, AAI secure vs insecure *r* = −0.003; *p* = 0.95; AAI dismissing vs preoccupied *r* = 0.02; *p* = 0.61), neither did the patients’ self-esteem at intake (Pearson Product Correlation Coefficient, AAI secure vs insecure *r* = 0.006; *p* = 0.89; AAI dismissing vs preoccupied *r* = −0.02; *p* = 0.59). Also, the therapists’ experience level did not correlate significantly with symptom severity at intake (*r* = −0.06; *p* = 0.16) or self-esteem at intake (*r* = 0.03; *p* = 0.44).

**FIGURE 1:**
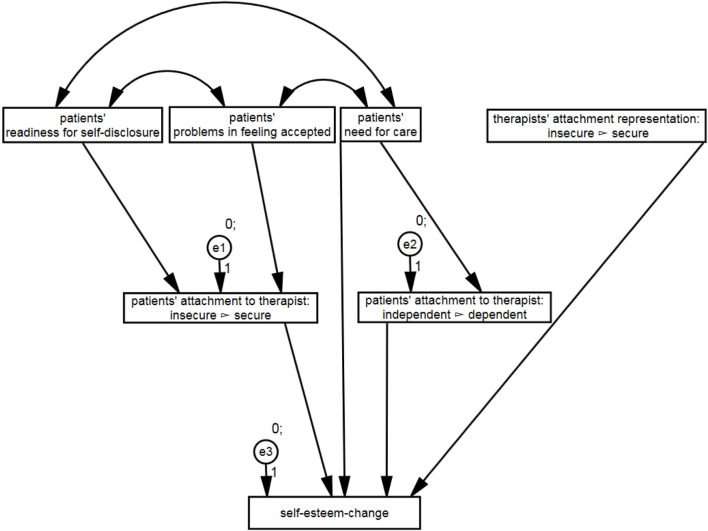
Structural relationships between patient’s and therapist’s attachment, attachment to the therapist and self-esteem-change. Only the significant paths are shown.

The aim of the study was to evaluate the differential effects of a patient’s and a therapist’s attachment on the attachment to the therapist at the end of the therapy and the impact of this bond on the self-esteem-change during therapy. The model depicted in Figure 1 fits the data quite well (see Table 3). All fit measures indicated a good model fit.

**TABLE 3:** Summary of fit indices.

*N*	Chi^2^ (df)	CMIN/DF	CFI	RMSEA	SRMR	TLI	NFI	AIC	ECVI
573	16.27 (7)	2.32	0.99	0.05	0.02	0.95	0.98	90.27	0.16

*df, degrees of freedom; CMIN/DF, minimum discrepancy, divided by its degrees of freedom; CFI, comparative fit index; RMSEA, root mean square error of approximation; TLI, Tucker Lewis Index; NFI, normed fit index; AIC, Akaike information criterion; ECVI, expected cross validation index.*

With regard to H1, the results showed that anticipated readiness for self-disclosure positively predicted the amount of perceiving the attachment to the therapist as secure: β = 0.33 (SE = 0.04), *p* < 0.001, standardized β = 0.33. Moreover, anticipated problems in feeling accepted negatively predicted the amount of perceiving the attachment to the therapist as secure: β = −0.25 (SE = 0.06), *p* < 0.001, standardized β = −0.24.

Regarding H2, results showed that anticipated need for care positively predicted the amount of perceiving the attachment to therapist as dependent: β = 0.29 (SE = 0.05), *p* < 0.001, standardized β = 0.33.

To sum up, hypotheses H1 and H2 could be confirmed.

Based upon the results, H3, stating that highly preoccupied therapists form attachment perceived as less dependent by the patient [β = 0.01 (SE = 0.02), *p* = 0.793, standardized β = 0.01] and that highly insecure therapists form attachment perceived as more dependent by the patient [β = −0.01 (SE = 0.02) *p* = 0.591, standardized β = −0.02] must be rejected.

Furthermore, hypotheses H4 could also be confirmed, stating that highly secure patients’ attachments to their therapists lead to greater changes in self-esteem: β = 11.91 (SE = 1.58), *p* < 0.001, standardized β = 0.33.

## Discussion

The present study was conducted to clarify the link between therapists’ and patients’ attachment styles as well as patients’ attachment to therapist and self-esteem change through psychotherapy. We hypothesized that the patients’ as well as the therapists’ attachment styles contribute to form an attachment relationship whose quality accounts for the amount of self-esteem-change.

We found a distribution pattern of attachment classification in therapists, assessed via AAI, slightly different to the distribution found by Dinger et al. (2009) or Schauenburg et al. (2010). Both studies showed mainly secure attachment patterns in therapists whereas in this study, a higher percentage of insecure attachment was found. The results showed that an individual patient’s attachment, measured by a questionnaire, had a reasonable influence on the attachment to their therapist. However, we found no significant influence of the therapist’s attachment representation, measured using the AAI, on the patient’s attachment to the therapist. Comparing different models, we could show that the amount of self-esteem-change depended rather on the actually formed attachment relationship between patients and therapists than on the respective attachment representations of the patients and therapists.

Previous research shows that high attachment security of the patient is associated with greater therapeutic alliance (e.g., Smith et al., 2010; Diener and Monroe, 2011; Levy et al., 2018; Levy and Johnson, 2019). In the present study, patients’ secure attachment is represented by high values in respect to readiness for self-disclosure and low values in respect to problems with feeling accepted. In accordance with the attachment theory of Bowlby (1982) and previous empirical work (Foster et al., 2007; Bylsma et al., 2010), the present results support the notion that readiness for self-disclosure and problems with feeling accepted are associated with the security perceived in the attachment to the therapist.

The result of an insignificant association between therapists’ attachment style and patients’ attachment to the therapist in the present study is in contrast to a study by Tyrrell et al. (1999) who found that securely attached therapists formed stronger therapeutic alliances. Moreover, the results do not support the suggestion, that the attachment relationship formed by highly preoccupied therapists is expected to be more dependent compared to that of more dismissive attached therapists (Bowlby, 1982), since no significant correlation between the therapists’ dismissive vs. preoccupied attachment status and the patients’ perception of dependency in the attachment to the therapist emerged. One reason for the absence of effects of the therapists’ attachment representation may be the small sample size of only 16 therapists.

To our knowledge, there exists no study, which examines the effect of attachment to the therapist on self-esteem-change in psychotherapy. The body of empirical research stated an association between secure attachment and stable high self-esteem (e.g., Foster et al., 2007; Fuhr et al., 2017). The present results even suggest that the experience of secure attachment to the therapist might stimulate a greater self-esteem-change. The hypotheses, that an attachment to the therapist perceived as highly secure and low dependent positively influences self-esteem change could be confirmed. In in-depth treatment self-esteem-change plays an important role in the process of therapy of different disorders as for example depression (Bettmann, 2006; Schaub et al., 2006; Fuhr et al., 2017), borderline personality disorder (Bateman and Fonagy, 2004; Stiglmayr and Gunia, 2016) or trauma (Gormley, 2004; König et al., 2012). At the same time, in an integrated overview, Berry and Danquah (2016) identified key goals and strategies of an attachment-informed psychotherapy for adults. Thereby, they summarized amongst other aspects that changing internal working models and creating a therapeutic relationship providing a secure base for patients is of importance. Nevertheless, there is to our knowledge no treatment combining both aspects by focusing on attachment during therapy to stimulate a greater self-esteem-change as therapy outcome. This aspect should be considered in therapeutical treatment by monitoring patients’ attachment to the therapist during therapy as it contributes to patients’ self-esteem. Additionally, it is also shown that readiness for self-disclosure and problems with feeling accepted are associated with the security perceived in the patients’ attachment to the therapist. As these are crucial factors for a successful therapeutic treatment, it supports our implication for at least monitoring attachment during therapy. In therapy process, aspects of strengthening the therapeutical alliance should be considered.

Although this study is based on a unique data set of patients with the respective treating therapists, there are limitations that must be considered. Even though the sample of patients is large, the results are based on a small sample of therapists, who mainly show an insecure attachment style in contrast to the findings by Dinger et al. (2009) or Schauenburg et al. (2010). Therefore, a replication of the results is required with a larger number of therapists treating a large number of patients. In addition, the evaluation of the attachment to the therapist depends on the therapist’s as well as the patient’s attachment; therefore, we do have a dependent data set. A multilevel analysis could be more appropriate to account for the dependency in our dataset with a nested structure–assessments as level-1, patients as level-2, and therapists as level-3. However, the dependent variable self-esteem was measured only twice (intake and discharge) in the present study and multilevel analysis is more suited to analyze change over more than two assessment points. In fact, three-level multilevel models did not converge with our data set when adding the intercept and/or the slope at the therapists-level as random effect(s). Furthermore, the attachment of the therapist and the patient was evaluated by different methods (questionnaire, interview). The use of an attachment questionnaire for the therapists was not considered since their knowledge and cognitive processes might have influenced the questionnaire ratings. Another solution would have been an attachment interview with all the patients. Since interviewing such a large sample of patients with the adult attachment interview was not manageable, the questionnaire was chosen. In a future study, an interview-based approach would be the best choice for investigating these hypotheses.

In future studies, the patients’ representation of the therapists’ using the Patient-Therapist AAI (PT AAI) by Diamond et al. (2003) might prove to be fruitful. The PT-AAI (Diamond, 2001; George et al., 2001) is a semi-structured interview developed as an adaptation of the AAI aimed at classifying the mental state concerning patients’ attachment to their therapists, and vice versa. Also, there are still numerous unanswered questions such as, for example, how counter-complementary attachment behavior can and should be used in therapeutic settings (e.g., Mallinckrodt, 2000). For clinical practice it would thus be of interest whether a response by the therapist to the patients’ attachment needs would further improve the understanding of the therapeutic process and increase in self-esteem. Further research on the interaction processes between the patients’ and the therapists’ attachment representations assessed by employing the same measures should be carried out in respect to other therapeutic outcome measures such as symptom reduction. In addition, the therapy drop-out rate needs to be examined more closely in reference to the patients’ attachment to the therapists as it might indicate mismatches and predict future drop-outs.

All in all, the present study shows effects of the patient’s attachment representations on the perceived attachment to the therapist as well as between the perceived attachment to the therapist and the amount of self-esteem-change. Nevertheless, the therapist’s attachment style had no significant influence on the perceived attachment to the therapist. Based on these results, it is assumed that self-esteem-change through psychotherapy is influenced by the actually formed attachment relationship as perceived by the patient. The patient’s attachment representations but not the therapist’s attachment style contributes to the actual patient’s attachment to the therapist. The mentioned clinical implications and approaches for future studies can provide deeper insides in the influence of attachment as well as attachment representations on the therapy process.

## Data Availability Statement

The data analyzed in this study is subject to the following licenses/restrictions: The dataset is a clinical dataset. Requests to access these datasets should be directed to KP, katja.petrowski@tu-dresden.de

## Ethics Statement

The studies involving human participants were reviewed and approved by Ethics Committee of the Technical University Dresden, Germany. The patients/participants provided their written informed consent to participate in this study.

## Author Contributions

KP designed the study, contributed to the data collection, and wrote the manuscript. HB, PB, VR, and TP contributed to the study design, data collection, and reviewed the final manuscript. All authors contributed to the article and approved the submitted version.

## Conflict of Interest

The authors declare that the research was conducted in the absence of any commercial or financial relationships that could be construed as a potential conflict of interest.

## Publisher’s Note

All claims expressed in this article are solely those of the authors and do not necessarily represent those of their affiliated organizations, or those of the publisher, the editors and the reviewers. Any product that may be evaluated in this article, or claim that may be made by its manufacturer, is not guaranteed or endorsed by the publisher.
